# Comparison of Optimal Machine Learning Algorithms for Early Detection of Unknown Hazardous Chemicals in Rivers Using Sensor Monitoring Data

**DOI:** 10.3390/toxics11040314

**Published:** 2023-03-27

**Authors:** Su Han Nam, Jae Hyun Kwon, Young Do Kim

**Affiliations:** 1 Department of Civil & Environmental Engineering, Myongji University, Yongin 17058, Republic of Korea; nsh3750@mju.ac.kr (S.H.N.);; 2 Department of Civil and Environmental Engineering, Nakdong River Basin Environmental Research Center, Inje University, Gimhae 50834, Republic of Korea

**Keywords:** machine learning, chemical contamination, alternative indicator, initial response, chemical detection

## Abstract

Water environment pollution due to chemical spills occurs constantly worldwide. When a chemical accident occurs, a quick initial response is most important. In previous studies, samples collected from chemical accident sites were subjected to laboratory-based precise analysis or predictive research through modeling. These results can be used to formulate appropriate responses in the event of chemical accidents; however, there are limitations to this process. For the initial response, it is important to quickly acquire information on chemicals leaked from the site. In this study, pH and electrical conductivity (EC), which are easy to measure in the field, were applied. In addition, 13 chemical substances were selected, and pH and EC data for each were established according to concentration change. The obtained data were applied to machine learning algorithms, including decision trees, random forests, gradient boosting, and XGBoost (XGB), to determine the chemical substances present. Through performance evaluation, the boosting method was found to be sufficient, and XGB was the most suitable algorithm for chemical substance detection.

## 1. Introduction

Chemical accidents in water environments have both natural and anthropogenic causes and refer to situations where large amounts of chemicals flow into rivers due to accidents occurring while handling or transporting chemicals [1]. Globally, chemical spills are a major cause of water pollution. Large-scale accidents causing water pollution occur regularly, such as the 1985 Old Delhi sulfuric acid spill in India, the 2004 Delaware River oil spill in the US, the 2005 sodium hydroxide spill in Canada, the 2007 Geelong River oil spill in France, the 2014 Arkansas River ferric sulfate spill in the US, and the 2015 sodium cyanide spill in Tianjin Port, China [2,3,4,5,6,7,8,9,10,11]. Despite the high rate of domestic river water use, industrial complexes in Republic of Korea are located near rivers. For example, the Nakdonggang is a major potable water source in the Yeongnam region, and large-scale industrial complexes are concentrated in the middle and upper regions of the river. Furthermore, chemical spills of substances such as phenols and 1,4-dioxane have caused pollution in the Nakdonggang [12,13].

Following a chemical spill, samples are typically collected in the field, and analyses are performed in the laboratory. Laboratory-based analyses are a common method for accurately detecting and quantifying pollutants. Various methods for analyzing chemical substances have been proposed, including those provided by the National Institute for Occupational Safety and Health, 1994; EPA ORD NHSRC, 2010a; EPA ORD NHSRC, 2010b; and OSHA Analytical Methods, 2022 [14,15,16,17]. In addition, Water Pollution Test Standards and Hazardous Chemical Test Standards have been established in Republic of Korea. Detecting the deterioration of water quality is critical for water conservation and public health [18]. Most methods are expensive and require specialized laboratories with sophisticated scientific equipment. In addition, highly qualified personnel are required to operate these devices [19].

In the event of a chemical accident, it is important to respond quickly to minimize the effect on aquatic ecosystems and humans. While petrochemical leaks can be observed with the naked eye, many chemicals that are likely to enter rivers are often colorless and water-soluble, making visual detection difficult. Furthermore, depending on its properties, a chemical substance may naturally decompose in the aqueous system; however, a non-reactive substance may remain in the system and cause secondary damage [20].

Studies on the transport and diffusion of oil spills have been performed using leaked-pollutant prediction [21,22,23], scenario simulation, and concentration prediction of chemical spills [24,25,26,27]. Additionally, data mining has been applied in several recent studies on leaked pollutants. To detect oil spills, Tong et al. used the random forest and self-similarity parameters [28], and Xu et al. used a support vector machine and local adaptive threshold [29]. Furthermore, Pelta et al. and Ozigis et al. analyzed oil spills using remote monitoring and machine learning (ML) [30,31]. Huang et al. used a support vector machine and conventional water quality sensors to detect pollutants [32], and Kwon et al. developed a framework that combines ML and a transient storage zone model to predict the location of chemical spills and pollutant mass [33].

Scenario analysis of chemical spills through modeling and data mining is important. However, the most critical aspect of chemical accidents is the early detection of leaks. Damage can be reduced by quickly determining the properties of the chemicals leaked into the river and implementing an appropriate response. In this study, chemical experiments were conducted using pH and electrical conductivity (EC), which are easy to measure in rivers. Thirteen types of chemicals were used in the chemical experiment. Chemical substances were measured from low to high concentrations, and pH and EC data were acquired according to concentration changes. Four ML algorithms were used: decision trees (DTs), random forests (RFs), gradient boosting (GB), and XGBoost (XGB). Finally, the results of the ML applications were compared to propose the most suitable algorithm for chemical substance detection. This study was conducted to better determine the optimal initial response in the event of chemical accidents using commonly used sensors, allowing quick and inexpensive detection of the spilled chemicals.

## 2. Materials and Methods

### 2.1. Chemical Reagents and Alternative Indicators

pH and EC were used as alternative indicators to determine chemical substances. A PP-50 pH meter (Sartorius AG, Göttingen, Germany) and YSI Pro 2030 (YSI, Yellow Springs, OH, USA) were used to measure the pH and EC, respectively.

Carbon-based organic materials have a low level of underwater dissociation, thereby limiting the measurements of pH and EC. In addition, since substances such as oil and phenol are easy to see with the naked eye when they enter a river, accidents are easy to detect, and countermeasures for these accident substances are already in place in Republic of Korea [1]. Therefore, 13 inorganic chemicals were selected in this study. The number of companies handling chemical substances in Republic of Korea, chemicals designated in various domestic and foreign agreements, and chemicals involved in actual chemical accidents were considered for the subject chemical substances. A total of 97 chemicals (accident preparedness substances) have been designated and managed by the Ministry of Environment in Republic of Korea as of 2018 after an investigation on the number of businesses handling each chemical substance [10]. Agreements on the use of chemicals are in place in not only Republic of Korea, but also globally, including the International Task Force (ITF-25), Extremely Hazardous Substances (EHSs) list, Chemical Weapons Convention (CWC), and Australia Group (AG). The ITF-25 regulates and manages 98 types of chemicals that can be used as chemical weapons for military purposes. In the US, 356 types of specific hazardous chemicals are designated and managed through the EHS to respond to environmental and safety risks caused by the storage and handling of toxic chemicals. The CWC designates and manages 42 types of toxic chemicals in 13 groups to control the development, production, acquisition, stockpiling, possession, and use of chemical weapons. Among the chemicals commonly manufactured and distributed in the private chemical industry, the AG controls imports and exports by identifying 63 kinds of chemicals that may be used to make chemical weapons. Table 1 shows the domestic and international designation status of the 13 chemicals selected for this study.

### 2.2. Selected Solvents

**Figure 1:**
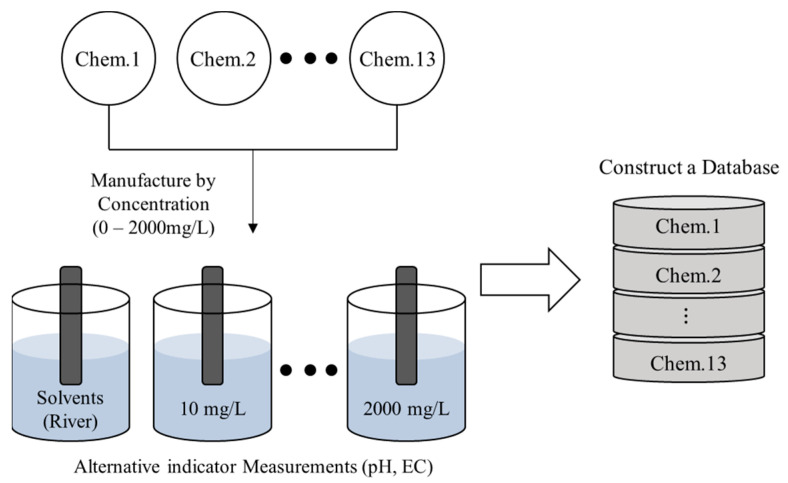
Schematic diagram of chemical (Chem.) database construction for pH and electrical conductivity (EC) measurements.

**Figure 2:**
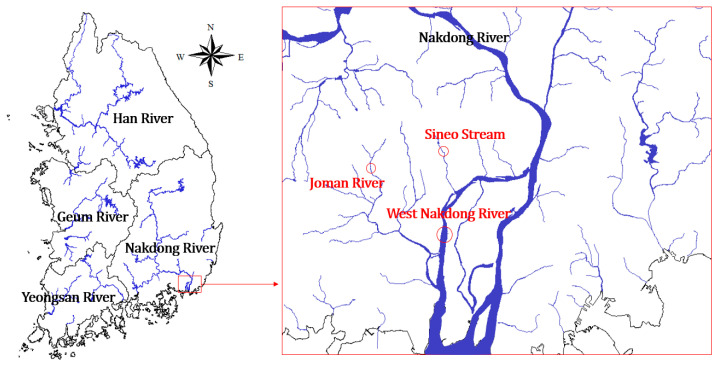
Water sampling points in Republic of Korea.

When high-concentration chemicals flow into a specific point in a river, they are transported and diffused along the flow direction. Experiments were conducted under the assumption that the high-concentration chemicals would be diluted over time by transport and diffusion (Figure 1). To avoid conducting experiments using chemicals in natural rivers, the water was sampled and used as a solvent (Figure 2). Additionally, rivers have different pH and EC base levels depending on their surrounding environments. Changes in these factors may vary depending on the characteristics of the river in the event of a chemical accident. Therefore, three rivers with different characteristics were selected and used as solvents for the chemical experiments (Table 2). These include Jomangang (JM), located in Juchon-myeon, Gimhae-si, Gyeongsangnam-do, which features agricultural and industrial areas around the water sampling point; Sineocheon (SE), which is a waterfront river in Gimhae-si, Gyeongsang-nam-do flowing through a residential area; and Seonakdonggang (SN) which is a lake-shaped river, wherein floodgates cause the water body to stagnate. The flow rate is controlled by the Noksan Floodgate in the estuary and the upstream Daejeo Floodgate [1].

### 2.3. Machine Learning

Classification algorithms in ML, a supervised learning technique, were applied in this study. By entering the measured alternative indicator as an input parameter, the corresponding chemical substance was produced as an output parameter. The applied ML algorithms were DTs, RFs, GB, and XGB.

DT is a machine learning method characterized by data searching and modeling [34], and both classification and regression models are used as nonparametric models [35]. Its advantage over other methods is that researchers can easily understand and interpret the analysis process [36]. DTs also require only a short time to develop and allows short-term predictions.

However, overfitting can easily occur in DTs; therefore, RFs, GB, and XGB were also compared and analyzed, and applied to an ensemble model to overcome this disadvantage. RF is a bagging ML algorithm that outputs classifications or average predictions from multiple DTs constructed during the training process, i.e., multiple DTs are created, and the outcome is determined by majority votes [37]. Each DT predictor for the RF is constructed by the random selection of samples and variables.

GB and XGB are boosting ML algorithms. Boosting combines inaccurate and weak learners to develop a more accurate and robust learner. The error resulting from a slightly inaccurate tree is compensated in the following tree to supplement the weakness before forming the next tree. GB achieves robust performance by combining weak learners to reduce the residuals; however, the method tends to overfit. XGB is an algorithm that is based on GB and adds a regularization term to the objective function to address this limitation. Compared with the existing GB method, XGB is a stepwise forward addition model and automatically utilizes multicore and distributed settings for an efficient learning process [38,39].

The ML algorithm was constructed using the chemical measurement database based on the alternative indicators. When establishing the ML algorithm, 80% of the overall data were used as training data and 20% as test data. While the training and test data were fixed datasets, the ML algorithm, which revises the hyperparameter using fixed data, only overfitted the fixed datasets; therefore, cross-validation was performed. In the cross-validation, the dataset was divided into ten parts, wherein one part was used to validate the effectiveness, while the others were used as a learning set to evaluate the ML algorithm [40]. All data were used to evaluate the applicability of the constructed ML algorithm for other datasets. All models and performance assessments were implemented using scikit-learn and XGBoost libraries in Python 3.9 (Python Software Foundation, Beaverton, OR, USA).

## 3. Results and Discussion

### 3.1. Alternative Indicator Measurement for the 13 Hazardous Chemical Substances

Table A1 shows the lab-scale experiment results of the alternative indicators (pH and EC) at each concentration of the 13 harmful chemical substances using three solvents (water from the Jomangang, Sineocheon, and Seonakdonggang). Measurements were carried out for 30 concentrations from 0 mg/L to 2000 mg/L. The pH values were affected by the base concentration of the rivers depending on the solvent, even when the chemical substance remained the same, resulting in differences in the low-concentration range. However, this difference gradually decreased to 100 mg/L. On the other hand, the EC was negligibly affected by the base concentration and constantly increased with the increase in the concentration for 11 of the 13 chemical substances, excluding bromine and arsenic trichloride. Therefore, the pH was affected by the base concentration of the chemicals in the rivers at low concentrations, whereas the EC was minimally affected.

Chemical substances with similar tendencies were classified into four groups based on their alternative indicator measurements (Table 3). Group 1 includes hydrochloric acid, ammonium difluoride, phosphorous pentafluoride, phosphorous pentasulfide, and ferric sulfate. As the concentration of these chemicals increased, the pH decreased and the EC increased. Furthermore, acidic properties were observed at 100 mg/L as the pH converged at approximately 2. Group 2 includes bromine and arsenic trichloride, which exhibited very little change in the EC, but a decrease in the pH as their concentrations increased. Group 3 includes potassium nitrate, potassium permanganate, and potassium chlorate. This group exhibited the opposite tendency of Group 2; i.e., for a given concentration change, the pH variation was very small, but the EC variation was significant. Finally, Group 4 includes sodium cyanide, potassium cyanide, and sodium hydroxide, which exhibited basic properties as both the pH and EC increased with changes in concentration.

The trends of the chemical substances were compared and classified by visualizing the changes in alternative indicators with their concentration. However, the individual detection of the 13 chemical substances was limited; therefore, ML was applied to detect the chemical substances. The ML algorithms DTs, RFs, GB, and XGB were compared and analyzed to select the optimal ML algorithm to detect the chemical substances.

### 3.2. Application of ML for Detecting the 13 Chemical Substances

**Figure 3:**
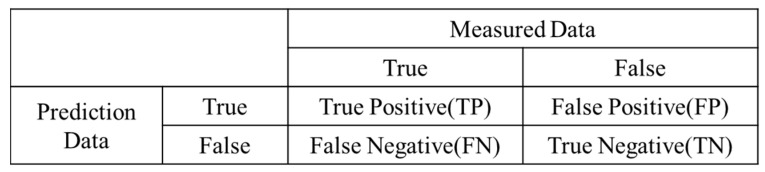
Confusion matrix (TP, FP, FN, and TN).

Hyperparameter tuning of DTs, RFs, GB, and XGB was performed, as shown in Table 4. Furthermore, a confusion matrix indicated whether the measured data agreed with the predicted data generated using the ML algorithms (Figure 3). The numbers in the confusion matrix represented the predicted results of the model. The detection and cross-validation results were evaluated in terms of accuracy. Receiver–operator characteristic (ROC) curves and AUC (area under the curve) were used to evaluate the performance of the four models. The ROC curve is a visual representation used to explain the diagnostic capability of the binary classifiers. The ROC curve reveals the sensitivity (true positive rate (TPR)) and specificity (1–falsefalse-positive rate (FPR)). Classifiers that provide curves closer to the top-left corner represent a reliable performance. As a baseline, a random classifier is required to place points along the diagonal line (FPR = TPR). When the curve reaches closer to the 45° diagonal of the ROC area, the test is less accurate [41].

(1) $$Accuracy=\frac{TP+TN}{TP+TN+FP+FN}$$

(2) $$True\;positive\;rate\left(TPR\right)=\frac{TP}{TP+FN}$$

(3) $$False\;positive\;rate\left(FPR\right)=\frac{FP}{TN+FP}$$

#### 3.2.1. Decision Tree

**Figure 4:**
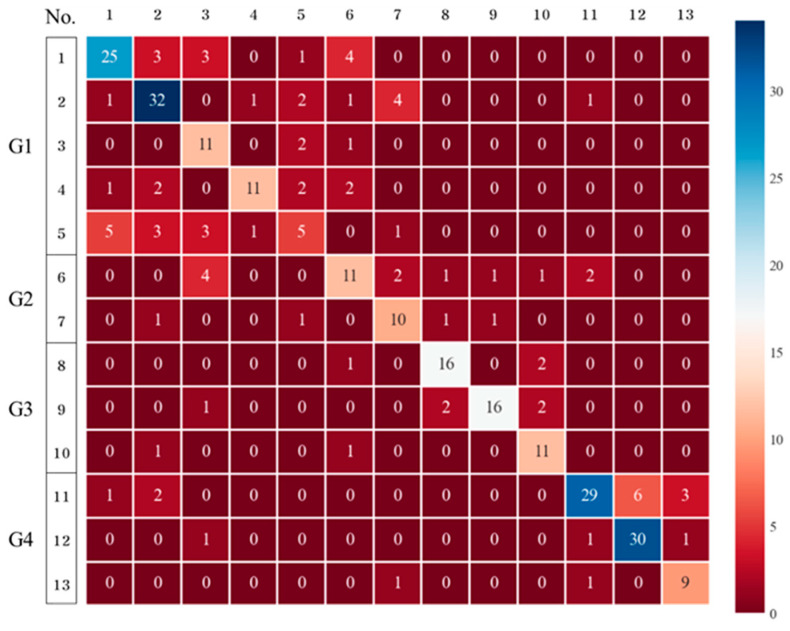
Confusion matrix (classification of the 13 chemicals using decision trees).

DTs are easy to understand and interpret and only require a short time to build, allowing for short-term predictions. The classification results of the 13 chemical substances determined by applying DTs are presented in a confusion matrix (Figure 4), which confirmed that chemical substances in the same group were also classified. The performance evaluation also showed a satisfactory result, with an accuracy of 0.7152. However, the deviation was between 0.5806 and 0.8333 in the cross-validation results, suggesting that it would be difficult to apply the constructed DT to other datasets. Therefore, the application of DTs for detecting the 13 chemical substances is limited.

#### 3.2.2. Random Forest

**Figure 5:**
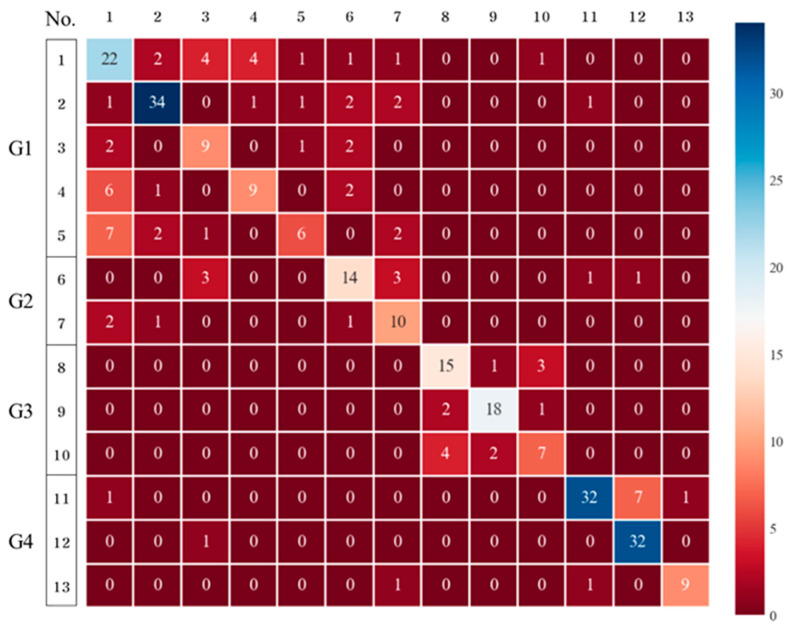
Confusion matrix (classification of the 13 chemicals using random forest).

The performance of RFs in detecting the chemical substances in Group 1 was lower than that of DTs, as shown in the confusion matrix in Figure 5. Additionally, the accuracy was 0.7185 in the evaluation performance, and the cross-validation results were 0.5667–0.8333, which is similar to those values of DTs. Therefore, the application of RFs is also limited.

#### 3.2.3. Gradient Boosting

**Figure 6:**
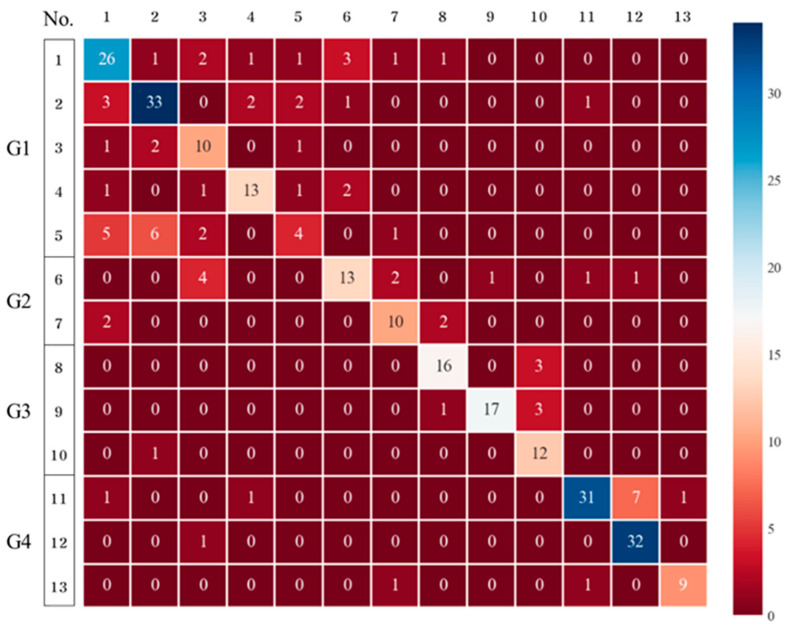
Confusion matrix (classification of the 13 chemicals using gradient boosting).

GB exhibited higher performance than DTs and RFs (accuracy = 0.7483) in the detection of the 13 chemical substances (Figure 6). Additionally, the cross-validation results were 0.6333–0.8065 and indicated no significant deviation, confirming the applicability of GB for different datasets. Therefore, GB was more suitable than the DT and bagging RF methods.

#### 3.2.4. XGBoost

**Figure 7:**
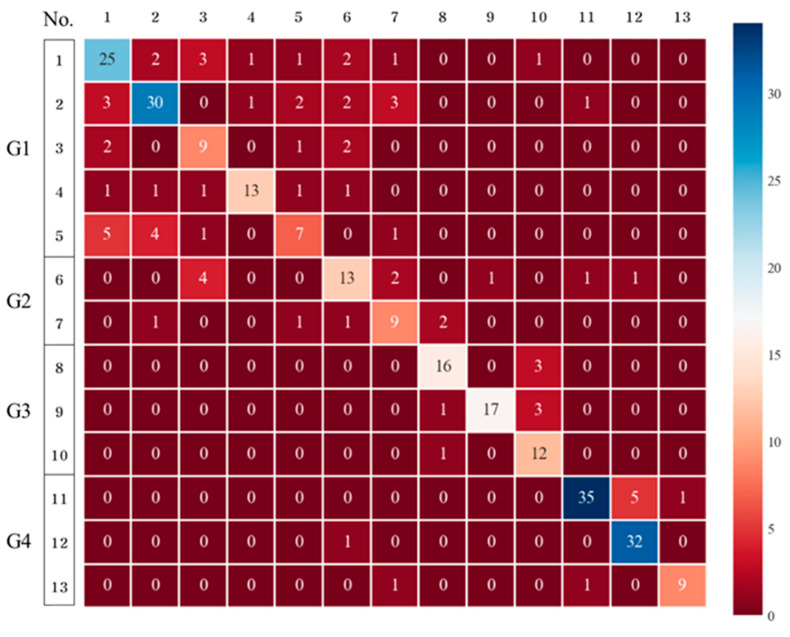
Confusion matrix (classification of the 13 chemicals using XGBoost).

The performance of XGB in the detection of the 13 chemical substances was higher than that of the GB method (Figure 7). The detection results for Groups 1–3 are similar to those of the DTs, RFs, and GB; however, the detection of Group 4 presented the best result. The performance evaluation also demonstrated the highest accuracy of 0.7517, and the deviations in the cross-validation results were also the smallest (0.6667–0.8000). Therefore, both boosting methods are sufficient for detecting the 13 chemical substances, with XGB being the most suitable.

To detect the 13 chemical substances, pH and EC were selected as alternative indicators to establish a database. The chemical substances with similar tendencies were classified based on the measurements of the alternative indicators according to changes in concentration; however, individual detection was limited, and therefore, ML was used to perform individual substance detection. Consequently, a significant deviation occurred when a different dataset was applied in the DT and RF methods, and the successful application of these methods was limited. The boosting methods, GB and XGB, exhibited a small deviation in the cross-validation results. Furthermore, XGB achieved the best performance in terms of accurately with detecting the 13 chemical substances, albeit only slightly. In summary, XGB is the most suitable ML algorithm for chemical detection. When using pH and EC as alternative indicators, the accuracy was 0.7-fold higher in most cases.

**Figure 8:**
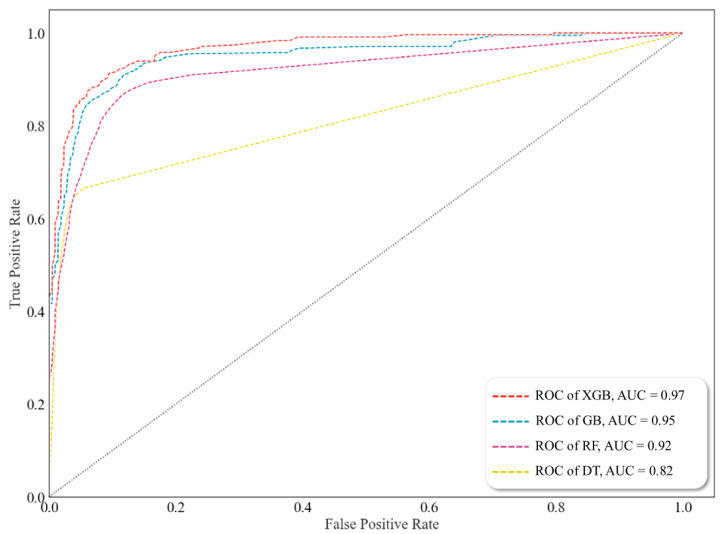
Accuracy evaluation for each machine learning algorithm using receiver operating characteristic (ROC) curve and area under the curve (AUC).

**Figure A1:**
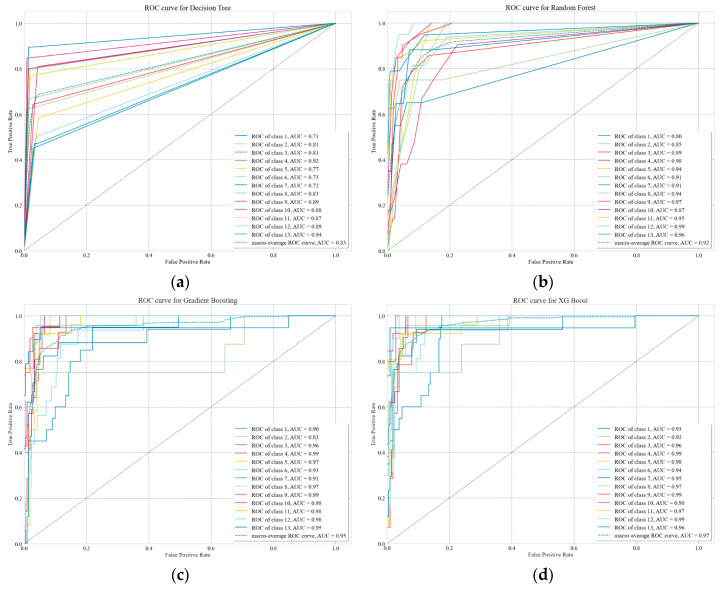
Accuracy evaluation for each machine learning algorithm using receiver operating characteristic curve and area under the curve. (**a**) ROC curve and AUC result of Decision Tree, (**b**) ROC curve and AUC result of Random Forest, (**c**) ROC curve and AUC result of Gradient Boosting, and (**d**) ROC curve and AUC result of XG Boost.

Figure 8 visualizes the ROCs for the four ML algorithms and shows the results of AUC calculations. Among the algorithms, XGB showed the best performance. Figure A1 shows the identification performance for the 13 chemical substances as ROC and AUC for each ML algorithm. The four algorithms successfully detected five chemicals (phosphorus pentasulfide, potassium permanganate, potassium chlorate, sodium cyanide, and potassium cyanide). The AUC of the DT (Figure A1a) was mostly below 0.9 and showed the lowest performance with an AUC of 0.83. For the RF (Figure A1b), which uses a bagging method, the detection performances for four chemicals (hydrogen chloride, ammonium bifluoride, phosphorus pentachloride, and potassium chlorate) were below 0.9, and the AUC was 0.92. GB (Figure A1c) and XGB (Figure A1d), which are boosting methods, showed a detection performance of 0.9 or higher for the 13 chemical substances. Among them, XGB showed the best detection performance for 12 out of 13 chemicals, not including sodium cyanide. Therefore, the boosting methods were the most suitable, and XGB was considered the most suitable ML algorithm for chemical detection.

## 4. Conclusions

In this study, changes in pH and EC caused by chemical substances entering three rivers in Republic of Korea in the event of a chemical accident were investigated. As direct detection of chemical substances is limited, a database of chemical concentrations was developed using pH and EC measurements as alternative indicators. Furthermore, the constructed database was applied to ML models to determine the best model for chemical detection.

The 13 types of chemical substances exhibited very similar tendencies when solvents of different base concentrations were used. There was a slight difference in pH at the low-concentration range, which was likely affected by the base concentration in the solvents. However, this difference decreased at ≥ 100 mg/L, and the effect of base concentration decreased in the high-concentration range. The effect of the base concentration on EC was minimal, and the EC of most chemical substances increased at a constant rate with increasing concentrations.

The chemical substances could be classified into four groups by visualizing the measurement results of the alternative indicators in response to substance concentrations where: Group 1 exhibited acidity; Group 2 exhibited decreased pH but showed little change in EC; Group 3 showed no change in pH, but an increase in EC; and Group 4 exhibited basic properties. The chemical substances could be detected in groups; however, the detection of individual chemical substances was limited; therefore, ML was applied.

The ML algorithms used in this study were DTs, RFs, GB, and XGB. Chemical substances in the same group could be detected using all four models. Moreover, all four models demonstrated a satisfactory accuracy of ≥0.7. The results of cross-validation varied among the models, with the DT and RF exhibiting considerable deviation. However, in boosting methods such as GB and XGB, the variance was found to be less pronounced in cross-validation. Based on these results the boosting methods were found to be more suitable than the DT and bagging methods. Among the boosting methods, XGB showed the highest accuracy in detecting chemical substances, making it the most appropriate method for this task.

In this study, a database of 13 chemical substances was created using alternative indicators. Furthermore, the chemical substances were detected using ML algorithms. Detecting chemical substances presents some challenges. However, this study provides a method that can quickly provide information about the leaked chemicals through an alternative index that can easily be measured in the field should a chemical accident occur. More precise and diverse chemical detection may be attained in the future by using various sensors and alternative indicators. The findings of this study can serve as basic data for developing an initial response to chemical accidents.

## Figures and Tables

**Table 1:** Selected hazardous chemicals managed by the International Task Force (ITF-25), Extremely Hazardous Substances (EHSs) list, Chemical Weapons Convention (CWC), Australia Group (AG), and Ministry of Environment (ME-97).

No.	Name	CAS No.	Number of Factories Using Chemical (Republic of Korea)	ITF-25	EHS	CWC	AG	ME-97 (Republic of Korea)
1	Hydrogen chloride	7647-01-0	3386	O	O	-	-	O
2	Ammonium bifluoride	1341-49-7	577	-	-	-	O	O
3	Phosphorus pentachloride	10026-13-8	15	-	O	O	O	O
4	Phosphorus Pentasulfide	1314-80-3	-	-	-	-	O	O
5	Ferric sulfate	10028-22-5	-	-	-	-	-	-
6	Bromine	7726-95-6	37	O	O	-	-	O
7	Arsenic trichloride	7784-34-1	-	O	O	O	O	O
8	Potassium nitrate	7757-79-1	185	-	-	-	-	-
9	Potassium permanganate	7722-64-7	167	-	-	-	-	-
10	Potassium chlorate	3811-04-9	19	-	-	-	-	-
11	Sodium cyanide	143-33-9	937	-	O	-	O	O
12	Potassium cyanide	151-50-8	265	-	O	-	O	O
13	Sodium hydroxide	1310-73-2	7998	-	-	-	-	-

**Table 2:** pH and EC values of selected solvents for rivers.

River	pH	EC (µS/cm)
Jomangang (JM)	7.5	335.6
Sineocheon (SE)	8.6	234.1
Seonakdonggang (SN)	8.7	350.2

**Table 3:** Group classification of the 13 chemicals.

Group	No.	Chemical
Group 1	1	Hydrogen chloride
	2	Ammonium bifluoride
	3	Phosphorus pentachloride
	4	Phosphorus pentasulfide
	5	Ferric sulfate
Group 2	6	Bromine
	7	Arsenic trichloride
Group 3	8	Potassium nitrate
	9	Potassium permanganate
	10	Potassium chlorate
Group 4	11	Sodium cyanide
	12	Potassium cyanide
	13	Sodium hydroxide

**Table 4:** Setting up machine learning (ML) hyperparameters for the detection of the 13 chemicals.

ML	Hyperparameter
Decision tree	Criterion = Gini, Max_depth = 50
Random forest	Criterion = Gini, Max_depth = 50, N_estimators = 10
Gradient boosting	Learning_rate = 0.15, Criterion = Fried_mse, Max_depth = 3, n_estimators = 70
XGBoost	Eta = 0.5, Max_depth = 20, N_estimators = 40, Minchild_weight = 1

## Data Availability

Not applicable.

## Appendix A

**Table A1:** Variation in pH and EC with concentration for several chemicals in surface water samples (log scale).

No.	Name	pH	EC
1	Hydrogen chloride		
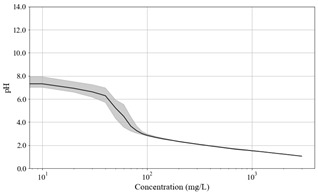
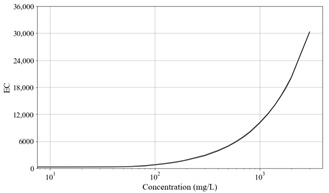
2	Ammonium bifluoride		
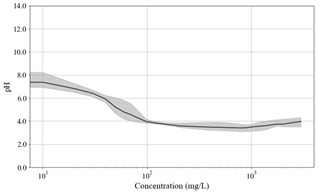
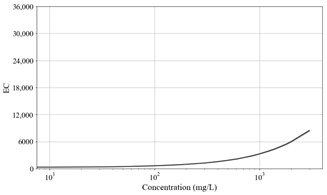
3	Phosphorus pentachloride		
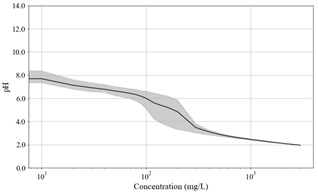
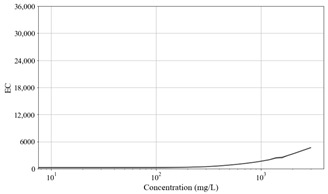
4	Phosphorus pentasulfide		
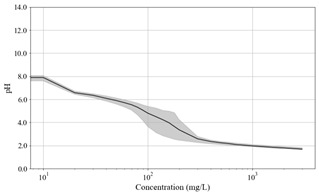
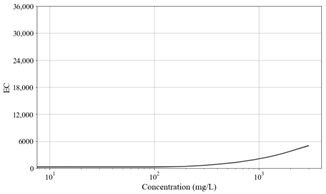
5	Ferric sulfate		
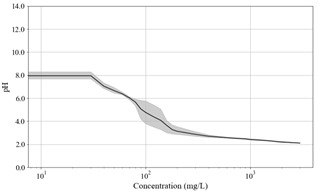
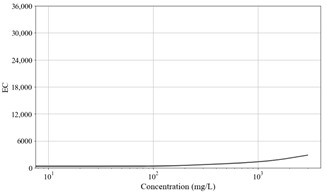
6	Bromine		
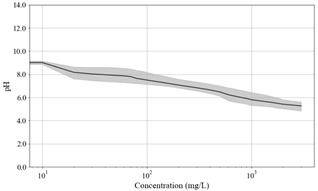
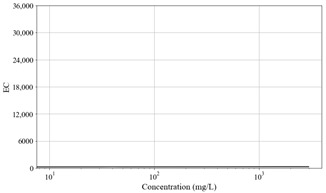
7	Arsenic trichloride		
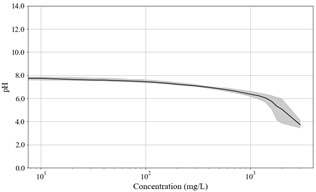
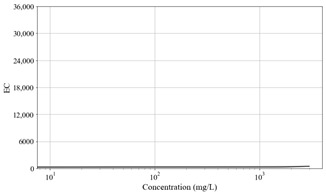
8	Potassium nitrate		
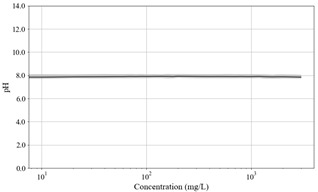
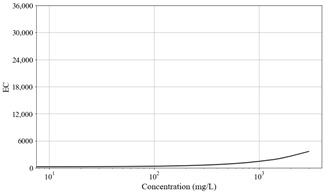
9	Potassium permanganate		
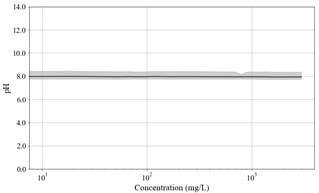
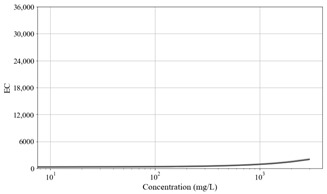
10	Potassium chlorate		
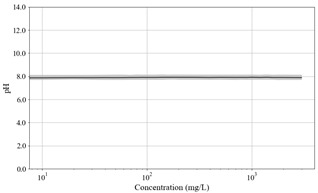
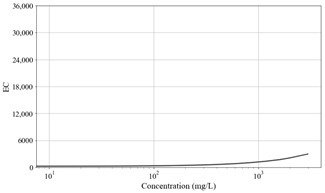
11	Sodium cyanide		
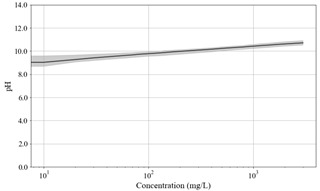
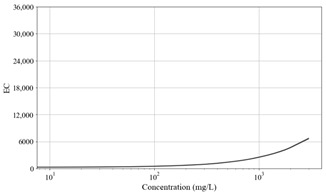
12	Potassium cyanide		
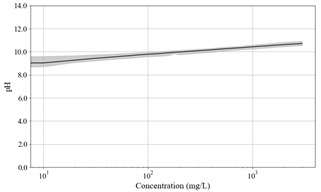
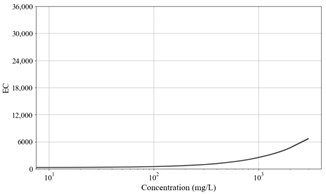
13	Sodium hydroxide		

## References

[1] Nam S.H., Ku T.G., Park Y.L., Kwon J.H., Huh D.S., Kim Y.D. (2022). Experimental study on the detection of hazardous chemicals using alternative sensors in the water environment. Toxics.

[2] Höfer T. (1998). Tainting of seafood and marine pollution. Water Res..

[3] Cordos E., Rautiu R., Roman C., Ponta M., Frentiu T., Sarkany A., Fodorpataki L., Macalik L., McCormick C., Weiss D. (2003). Characterization of the rivers in the mining and industrial area of Baia Mare, Romania. Eur. J. Min. Process. Environ. Protect..

[4] Alonso E., Santos A., Callejón M., Jiménez J.C. (2004). Speciation as a screening tool for the determination of heavy metal surface water pollution in the Guadiamar river basin. Chemosphere.

[5] McArthur M., Wind E. (2007). Amphibian Assessment Following the Accidental Release of Sodium Hydroxide into the Cheakamus River, British Columbia. https://www.researchgate.net/publication/242113199.

[6] Gangopadhyay R.K., Das S.K. (2008). Lessons learned from a fuming sulfuric acid tank overflow incident. J. Chem. Health Saf..

[7] Hou Y., Zhang T.Z. (2009). Evaluation of major polluting accidents in China—Results and perspectives. J. Hazard. Mater..

[8] Cabon J.Y., Giamarchi P., Le Floch S. (2010). A study of marine pollution caused by the release of metals into seawater following acid spills. Mar. Pollut. Bull..

[9] Zunkel A., Tiebe C., Schlischka J. (2014). “Stolt Rotterdam”–The sinking of an acid freighter. Eng. Fail. Anal..

[10] Ministry of Environment (ME) (2022). Chemical Substance Statistics Disclosure. https://icis.me.go.kr/pageLink.do.

[11] Hou J., Gai W.M., Cheng W.Y., Deng Y.F. (2021). Hazardous chemical leakage accidents and emergency evacuation response from 2009 to 2018 in China: A review. Saf. Sci..

[12] Lee K.S. (2011). Drinking Water Resource Projects in Gyeongbuk and Daegu.

[13] Choi M.O. (2013). A case study of environmental policy formation: A focus on the phenol spills in Nakdong River of 1991 and 2008. GRI Rev..

[14] National Institute for Occupational Safety and Health (1994). NIOSH Manual of Analytical Methods.

[15] EPA, ORD, NHSRC (2010). Rapid Screening and Preliminary Identification Techniques and Method. EPA/600/R-10/090. https://cfpub.epa.gov/si/si_public_record_report.cfm?dirEntryId=227244&Lab=NHSRC.

[16] EPA, ORD, NHSRC (2010). Sample Collection Information Document for Pathogens and Biotoxins. EPA/600/R-09/074. https://19january2017snapshot.epa.gov/homeland-security-research/sample-collection-information-document-pathogens-and-biotoxins-companion_.html.

[17] OSHA Analytical Methods. https://www.osha.gov/chemicaldata/sampling-analytical-methods.

[18] Sambito M., Freni G. (2021). Strategies for improving optimal positioning of quality sensors in urban drainage systems for non-conservative contaminants. Water.

[19] Yaroshenko I., Kirsanov D., Marjanovic M., Lieberzeit P.A., Korostynska O., Mason A., Frau I., Legin A. (2020). Real-time water quality monitoring with chemical sensors. Sensors.

[20] Gwon Y., Kim D., You H. (2020). A standardized procedure on building spectral library for hazardous chemicals mixed in river flow using hyperspectral image. J. Korea Water Resour. Assoc..

[21] Dunsbergen D.W., Stalling G.S. (1970). The combination of a random walk method and a hydrodynamic model for the simulation of dispersion of dissolved matter in water. WIT Trans. Ecol. Environ..

[22] Wang S.D., Shen Y.M., Guo Y.K., Tang J. (2008). Three-dimensional numerical simulation for transport of oil spills in seas. Ocean Eng..

[23] Craig P.M. (2009). Implementation of a Lagrangian Particle Tracking Sub-Model for the Environmental Fluid Dynamics Code.

[24] Neely W.B., Blau G.E., Alfrey T. (1976). Mathematical models predict concentration-time profiles resulting from chemical spill in a river. Environ. Sci. Technol..

[25] Fu W., Fu H., Skøtt K., Yang M. (2008). Modeling the spill in the Songhua River after the explosion in the petrochemical plant in Jilin. Environ. Sci. Pollut. Res..

[26] Bahadur R., Samuels W.B. (2015). Modeling the fate and transport of a chemical spill in the Elk River, West Virginia. J. Environ. Eng..

[27] Yeom J., Kim I., Kim M., Cho K., Kim S.D. (2020). Coupling of the AQUATOX and EFDC models for ecological impact assessment of chemical spill scenarios in the Jeonju River, Korea. Biology.

[28] Tong S., Liu X., Chen Q., Zhang Z., Xie G. (2019). Multi-feature based ocean oil spill detection for polarimetric SAR data using random forest and the self-similarity parameter. Remote Sens..

[29] Xu J., Wang H., Cui C., Zhao B., Li B. (2020). Oil spill monitoring of shipborne radar image features using SVM and local adaptive threshold. Algorithms.

[30] Ozigis M.S., Kaduk J.D., Jarvis C.H. (2019). Mapping terrestrial oil spill impact using machine learning random forest and Landsat 8 OLI imagery: A case site within the Niger Delta region of Nigeria. Environ. Sci. Pollut. Res..

[31] Pelta R., Carmon N., Ben-Dor E. (2019). A machine learning approach to detect crude oil contamination in a real scenario using hyperspectral remote sensing. Int. J. Appl. Earth Obs. Geoinf..

[32] Huang P., Jin Y., Hou D., Yu J., Tu D., Cao Y., Zhang G. (2017). Online classification of contaminants based on multi-classification support vector machine using conventional water quality sensors. Sensors.

[33] Kwon S., Noh H., Seo I.W., Jung S.H., Baek D. (2021). Identification framework of contaminant spill in rivers using machine learning with breakthrough curve analysis. Int. J. Environ. Res. Public Health.

[34] Linoff G.S., Berry M.J. (2011). Data Mining Techniques: For Marketing, Sales, and Customer Relationship Management.

[35] Breiman L. (1996). Bagging predictors. Mach. Learn..

[36] Cho Y., Kim Y.C., Shin Y. (2017). Prediction model of construction safety accidents using decision tree technique. J. Korea Inst. Build. Constr..

[37] Jung W.S., Kim S.E., Kim Y.D. (2021). Analysis of influential factors of cyanobacteria in the mainstream of Nakdong river using random forest. J. Wetl. Res..

[38] Mitchell R., Frank E. (2017). Accelerating the XGBoost algorithm using GPU computing. PeerJ Comput. Sci..

[39] Zhang H., Si S., Hsieh C.J. (2017). GPU-acceleration for large-scale tree boosting. arXiv.

[40] Raschka S. (2018). Model evaluation, model selection, and algorithm selection in machine learning. arXiv.

[41] Al-Azzam N., Shatnawi I. (2021). Comparing supervised and semi-supervised machine learning models on diagnosing breast cancer. Ann. Med. Surg..

